# Galactose-Deficient IgA1 as a Candidate Urinary Marker of IgA Nephropathy

**DOI:** 10.3390/jcm11113173

**Published:** 2022-06-02

**Authors:** Yusuke Fukao, Hitoshi Suzuki, Jin Sug Kim, Kyung Hwan Jeong, Yuko Makita, Toshiki Kano, Yoshihito Nihei, Maiko Nakayama, Mingfeng Lee, Rina Kato, Jer-Ming Chang, Sang Ho Lee, Yusuke Suzuki

**Affiliations:** 1 Department of Nephrology, Faculty of Medicine, Juntendo University, Tokyo 113-8421, Japan; y-fukao@juntendo.ac.jp (Y.F.); ymakita@juntendo.ac.jp (Y.M.); tkano@juntendo.ac.jp (T.K.); y-nihei@juntendo.ac.jp (Y.N.); m-nakayama@juntendo.ac.jp (M.N.); m-ri@juntendo.ac.jp (M.L.); r-fujikawa@juntendo.ac.jp (R.K.); 2 Department of Nephrology, Juntendo University Urayasu Hospital, Chiba 279-0021, Japan; 3 Division of Nephrology, Department of Internal Medicine, College of Medicine, Kyung Hee University, Seoul 130-701, Korea; jinsuk0902@naver.com (J.S.K.); kyunghwan@naver.com (K.H.J.); lshkidney@khu.ac.kr (S.H.L.); 4 Division of Nephrology, Kaohsiung Medical University Hospital, Kaohsiung 80756, Taiwan; jemich@cc.kmu.edu.tw

**Keywords:** urinary galactose-deficient IgA1, KM55, IgA nephropathy

## Abstract

In patients with IgA nephropathy (IgAN), circulatory IgA1 and IgA1 in the mesangial deposits contain galactose-deficient IgA1 (Gd-IgA1). Some of the Gd-IgA1 from the glomerular deposits is excreted in the urine and thus urinary Gd-IgA1 may represent a disease-specific marker. We recruited 338 Japanese biopsy-proven IgAN patients and 120 patients with other renal diseases (disease controls). Urine samples collected at the time of renal biopsy were used to measure Gd-IgA1 levels using a specific monoclonal antibody (KM55 mAb). Urinary Gd-IgA1 levels were significantly higher in patients with IgAN than in disease controls. Moreover, urinary Gd-IgA1 was significantly correlated with the severity of the histopathological parameters in IgAN patients. Next, we validated the use of urinary Gd-IgA1 levels in the other Asian cohorts. In the Korean cohort, urinary Gd-IgA1 levels were also higher in patients with IgAN than in disease controls. Even in Japanese patients with IgAN and trace proteinuria (less than 0.3 g/gCr), urinary Gd-IgA1 was detected. Thus, urinary Gd-IgA1 may be an early disease-specific biomarker useful for determining the disease activity of IgAN.

## 1. Introduction

Immunoglobulin A nephropathy (IgAN) is the most common primary glomerulonephritis [1]. According to a systematic review of 40 worldwide studies, the incidence of IgAN is reportedly 2.5/100,000/year [2]. If left untreated, IgAN has a poor prognosis, developing into end-stage renal failure in approximately 20% to 40% of cases within 20 years after onset [3].

There are two IgA isotypes in humans, IgA1 and IgA2 [4]. Galactose-deficient IgA1 (Gd-IgA1), which lacks galactose (Gal) in the O-glycan side chains in the hinge region and exposes N-acetylgalactosamine (GalNAc), has been identified as one of the key molecules in the pathogenesis of IgAN and is increased in the sera of patients with IgAN [5]. According to the multi-hit hypothesis [6], Gd-IgA1 is recognized by anti-glycan autoantibodies, resulting in the formation of pathogenic immune complexes. These immune complexes are deposited in the kidneys, activate mesangial cells, and induce glomerular injury.

Serum Gd-IgA1 levels can predict IgAN progression [7,8]. In contrast, most relatives of IgAN patients with abnormal IgA1 glycoforms do not develop IgAN [9], and serum Gd-IgA1 levels do not correlate with proteinuria [5], suggesting that serum Gd-IgA1 may not be a useful biomarker.

We previously developed a monoclonal antibody (KM55 mAb) that specifically recognizes Gd-IgA1, and demonstrated that glomerular Gd-IgA1 was specifically detected in IgAN and IgA vasculitis by immunohistochemical analysis using KM55 mAb [10,11].

In a mouse model, injection of purified nephritogenic IgA from IgAN-prone mice led to deposition in the glomeruli in nude mice. Parts of the injected IgA passed through into the bladder, suggesting that some parts of such glomerular IgA were cleared into the urine. In addition, the nephritogenic IgA had strong affinity not only to the glomerular mesangium, but also to the subepithelium [12]. On the other hand, several studies have supported that epithelial cells have the potential to clear matrix material and epithelial deposits into the cavity of Bowman’s capsule [13]. Therefore, we hypothesized that urinary Gd-IgA1 could be a disease-specific marker. Indeed, an enzyme-linked immunosorbent assay (ELISA) using Helix aspersa agglutinin (HAA), a GalNAc-specific lectin, could detect urinary Gd-IgA1 and differentiate patients with IgAN from patients with other renal diseases [14]. However, lectin-dependent assays are not suitable for large-scale, multi-specimen testing because of the instability of glycan recognition. Thus, a novel and stable assay is required for the early detection of IgAN.

Many studies have shown that the degree of proteinuria is an outcome predictor in IgAN [15,16]. However, it is difficult to determine whether urinary protein excretion is due to active lesions triggered by glomerular immune deposition or chronic lesions represented by glomerulosclerosis and nephron reduction [17]. Therefore, proteinuria may not always reflect the disease activity, and its assessment is insufficient to determine the indications for treatment.

Several reports have indicated a high remission rate of tonsillectomy combined with steroid pulse therapy in the early stages of IgAN [18,19]. In addition, the renal biopsy findings of 56 patients with hematuria without overt proteinuria revealed that IgAN was common in their pathological diagnoses, and 31% of the patients with IgAN had crescentic lesions [20]. Thus, early diagnosis and treatment of IgAN are important for remission, and a useful biomarker for the indication of renal biopsy is desired.

There are no established disease-specific biomarkers for IgAN. Furthermore, repeated renal biopsies are difficult because of the invasiveness. We established a stable and simple ELISA for Gd-IgA1 using the KM55 mAb in 2015 [11]. In this study, we investigated the usefulness of urinary Gd-IgA1 as a disease-specific marker for IgAN from Japanese cohorts and further verified this using non-Japanese Asian cohorts.

## 2. Materials and Methods

### 2.1. Patients and Samples

We recruited 338 Japanese adults (≥18 years old) with biopsy-proven IgAN and 120 patients with other renal diseases (disease controls) diagnosed at Juntendo University Hospital, Tokyo, Japan from 2015 to 2018. In addition, to validate the use of the urinary Gd-IgA1 level, we recruited 69 Korean and 35 Taiwanese biopsy-proven IgAN patients, as well as 39 Korean disease control patients.

Clinical and laboratory data were collected at the time of the renal biopsy. The laboratory parameters included serum creatinine (Cr) levels, serum Gd-IgA1 levels, urinary protein-to-creatinine ratios (UPCR), and urinary Gd-IgA1 levels.

### 2.2. Pathological Parameters

The histological samples were classified according to the clinical guidelines for IgAN from the Japanese Society of Nephrology (JSN) [21] or the Oxford classification [22,23]. Briefly, the histological grade (H-grade) of the JSN criteria was defined as 1 (0–24.9%), 2 (25–49.9%), 3 (50–74.9%), and 4 (75–100%) based on the percentage of glomeruli with pathological features, such as crescents, global sclerosis, and segmental sclerosis, which predict the progression to end-stage renal disease.

### 2.3. Measurement of Gd-IgA1

Serum and urinary Gd-IgA1 levels were determined using the KM55 mAb according to the manufacturer’s instructions (Immuno-Biological Laboratories, Fujioka, Japan), and logarithmically transformed (log10 basis).

### 2.4. Statistical Analyses

Data are expressed as mean ± standard error. Comparisons between groups were performed using the Mann–Whitney U test. Spearman’s correlation analysis was used to analyze the correlation between two variables. Statistical significance was defined as *p* < 0.05. Statistical analyses were performed using GraphPad Prism software ver.8.0 (GraphPad Software, San Diego, CA, USA).

## 3. Results

### 3.1. Demographic, Clinical, and Laboratory Findings

The clinical characteristics of the cohorts from each country at the time of renal biopsy are presented in Table 1. In the Japanese and Korean cohorts, there were no significant differences in age or sex between patients with IgAN and the disease controls. Compared to Japanese patients with IgAN, Korean patients showed higher levels of proteinuria and lower kidney function at the time of renal biopsy.

Other renal diseases included lupus nephritis, anti-neutrophil cytoplasmic antibody (ANCA)-associated glomerulonephritis, membranous nephropathy, focal segmental glomerulosclerosis, minimal change disease, membranoproliferative glomerulonephritis, non-IgA mesangial proliferative glomerulonephritis, tubulointerstitial nephritis, diabetic kidney disease, renal amyloidosis, nephrosclerosis, and thin basement membrane disease (Table 2).

### 3.2. Urinary Levels of Gd-IgA1 in the Japanese Cohort

**Figure 1:**
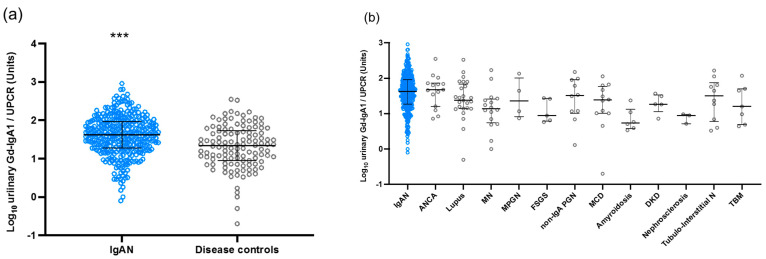
Urinary levels of Gd-IgA1 in Japanese cohort. (**a**) Urinary Gd-IgA1 levels were significantly elevated in patients with IgAN compared to those in disease controls. (**b**) Urinary Gd-IgA1 levels were lower in the disease controls than in IgAN patients. *** *p* < 0.0001.

In the Japanese cohort, urinary Gd-IgA1 levels were significantly elevated in patients with IgAN compared to those in disease controls (*p* < 0.0001) (Figure 1a). The levels of urinary Gd-IgA1 in each disease control group are shown in Figure 1b. Overall, urinary Gd-IgA1 levels were lower in the disease controls than in IgAN patients.

### 3.3. Correlation between Urinary Gd-IgA1 and Laboratory and Pathological Findings in IgAN Patients from the Japanese Cohort

**Figure 2:**
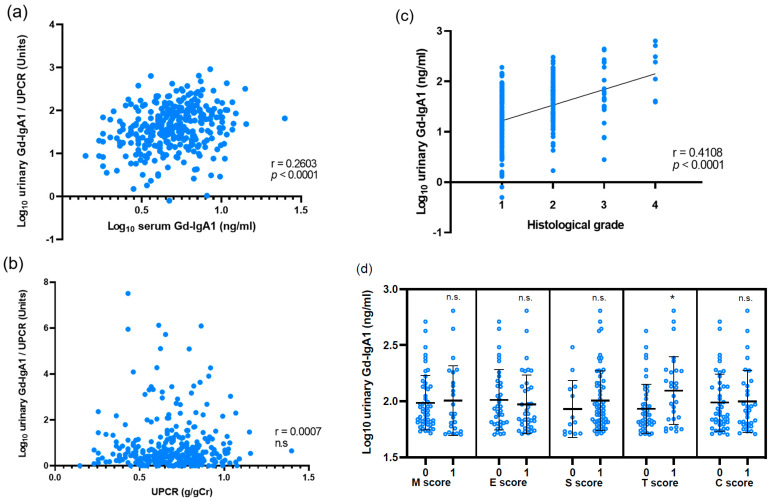
Correlation between urinary Gd-IgA1 and laboratory and pathological findings in IgAN patients from the Japanese cohort. (**a**) The levels of urinary Gd-IgA1 associated with the levels of serum Gd-IgA1. (**b**) The levels of urinary Gd-IgA1 did not correlate with the levels of proteinuria. (**c**) Urinary Gd-IgA1 levels were positively correlated with the histological grade. (**d**) Urinary Gd-IgA1 levels were positively correlated with the T score of the Oxford classification in cases with urinary Gd-IgA1 greater than 50 ng/mL. * *p* < 0.01, n.s.: not significant, Abbreviations: M (mesangial hypercellularity); E (endocapillary hypercellularity); S (segmental glomerulosclerosis); T (tubular atrophy/interstitial fibrosis); and C (cellular/fibrocellular crescents).

We assessed the association between urinary Gd-IgA1 levels and the clinical data and pathological parameters in patients with IgAN from the Japanese cohort. The levels of urinary Gd-IgA1 were associated with the levels of serum Gd-IgA1 (*p* < 0.0001) (Figure 2a), but not with proteinuria, in patients with IgAN (Figure 2b). As shown in Figure 2c, urinary Gd-IgA1 levels were positively correlated with the histological grade (*R* = 0.4108, *p* < 0.0001). Meanwhile, there were no significant correlations between the urinary Gd-IgA1 levels and the MEST-C scores of the Oxford classification. Then, we placed a threshold on the urinary Gd-IgA1 levels, i.e., >50 ng/mL, and analyzed the association between urinary Gd-IgA1 levels and MEST-C Oxford classification. As shown in Figure 2d, there were significant correlations between the urinary Gd-IgA1 levels and the T score of the Oxford classification in cases with urinary Gd-IgA1 greater than 50 ng/mL (*p* < 0.01).

### 3.4. Validation in Korean Cohort

**Figure 3:**
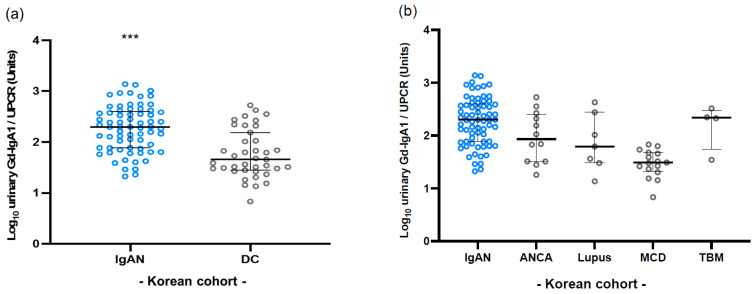
Urinary levels of Gd-IgA1 in Korean and Taiwanese cohorts. (**a**) The levels of urinary Gd-IgA1 in Korean patients with IgAN were significantly higher compared with those in disease controls. (**b**) The levels of urinary Gd-IgA1 in Korean patients with IgAN were higher than those in the any other disease controls. *** *p* < 0.0001.

Next, we validated the use of urinary Gd-IgA1 levels for the diagnosis of IgAN using the Korean cohort. In the Korean cohort, the levels of urinary Gd-IgA1 in patients with IgAN were significantly higher compared with those in disease controls (*p* < 0.0001) (Figure 3a). Moreover, the levels of urinary Gd-IgA1 in patients with IgAN were higher than those in any of the disease controls (Figure 3b).

### 3.5. Difference in Clinical Features at the Time of Renal Biopsy in Patients with IgAN

Compared with the Japanese cohort, Korean and Taiwanese patients showed greater levels of proteinuria and lower kidney function at the time of renal biopsy (Table 3). In addition, levels of urinary Gd-IgA1 in the Korean and Taiwanese patients with IgAN were higher than those in the Japanese patients (Supplementary Figure S1).

### 3.6. Urinary Gd-IgA1 Excretion with Trace Proteinuria

**Figure 4:**
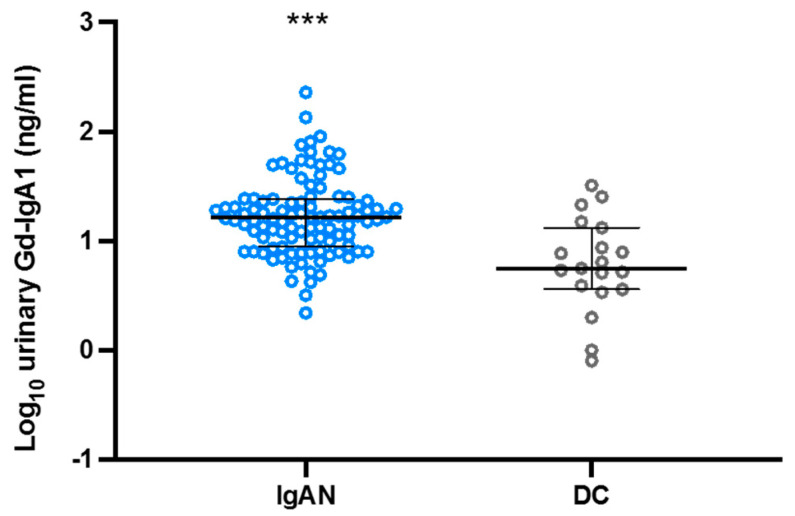
Urinary Gd-IgA1 excretion in cases with trace amounts of proteinuria (less than 0.3 g/gCr). *** *p* < 0.0001.

We assessed the urinary Gd-IgA1 excretion in cases with trace proteinuria (less than 0.3 g/gCr) using the Japanese cohort. Even in cases with trace proteinuria, urinary Gd-IgA1 was detected, and the levels of urinary Gd-IgA1 were higher in patients with IgAN than in disease controls (Figure 4).

## 4. Discussion

A definitive diagnosis of IgAN requires a pathological diagnosis via renal biopsy. In Japan, an annual screening for urinary abnormalities is performed in school-aged children, and asymptomatic patients with microscopic hematuria or mild proteinuria are more likely to undergo renal biopsy than are patients in other countries [24,25]. Indications for renal biopsy vary in each country due to medical insurance. Clinical remission can be achieved with an early diagnosis and treatment [26]. Therefore, a useful biomarker for early detection is required.

Gd-IgA1 is a critical effector molecule in the pathogenesis of IgAN. Glomerular Gd-IgA1 has been specifically detected in patients with IgAN but not in those with other renal diseases [10]. A previous experiment using real-time imaging revealed that injected IgA from the serum of IgAN-prone mice bound to the glomeruli in normal mice, and these IgA deposits cleared over time [12]. Thus, we hypothesized that a fraction of the Gd-IgA1 in glomerular deposits may be excreted into the urine. Previous reports using HAA lectins supported this hypothesis [14]. In this study, we used a stable lectin-independent method with the KM55 mAb to measure the urinary levels of Gd-IgA1. Indeed, the urinary excretion of Gd-IgA1 was elevated in patients with IgAN compared with that in disease controls. This may be due to a mechanism in IgAN in which the greater the amount of Gd-IgA1 deposited, the more glomerular injury occurs, resulting in the increased excretion of urinary Gd-IgA1. As reported in a systematic review [27], the usefulness of serum Gd-IgA1 levels as a tool for assessing disease severity is controversial. In the present study, we found a correlation between urinary Gd-IgA1 levels and the histological severity in IgAN patients. Moreover, the T score of the Oxford classification was associated with urinary Gd-IgA1 levels. Previous reports indicated that the T score is associated with a poor renal outcome [28,29]. Of note, the T score was significantly associated with the renal outcome, independently of clinical data [30]. This suggests that urinary Gd-IgA1 may be useful in determining the disease activity of IgAN.

IgAN is a multifactorial disease with a complex pathogenesis involving genetic and environmental factors. Therefore, we validated the urinary Gd-IgA1 assay in different cohorts of IgAN patients. The levels of urinary Gd-IgA1 were also significantly higher in patients with IgAN than in disease controls in the Korean cohort. Of note, in the Taiwanese and Korean cohorts, urinary Gd-IgA1 levels were much higher than those in the Japanese cohort, which may be due to differences in indications for renal biopsy in these countries, resulting in a higher proportion of advanced-stage cases with a higher urinary protein level and lower eGFR at the time of the renal biopsy. 

Clinically, a renal biopsy is rarely performed if proteinuria is negative, even in patients with hematuria. A single-center study in Japan reported that as many as 31% of IgAN patients with hematuria without overt proteinuria (less than 0.3 g/gCr) showed crescents in the renal biopsy findings [20]. Moreover, even in the early stages of IgAN, with normal kidney function and trace proteinuria, long-term renal survival is not always favorable [31]. As early identification and treatment can lead to clinical remission of IgAN [18], biomarkers that can be used for early diagnosis and enable determination of the timing of therapeutic interventions are needed. In the present study, even in patients with IgAN and trace proteinuria (less than 0.3 g/gCr), urinary Gd-IgA1 was detected. Importantly, this suggests that urinary Gd-IgA1 is an early biomarker compared to proteinuria in patients with IgAN.

There are several limitations in the present study. First, we only included Asian patients with IgAN. Large-scale, worldwide studies are needed to elucidate the underlying mechanisms of urinary Gd-IgA1 excretion. Moreover, longitudinal analysis during course of treatment is desired to elucidate disease activity.

In summary, we found higher urinary levels of Gd-IgA1 in patients with IgAN than in patients with other renal diseases. Urinary Gd-IgA1 may be a highly disease-specific marker in several Asian countries. Furthermore, urinary Gd-IgA1 can be detected in patients with trace proteinuria. The present study suggests that urinary Gd-IgA1 is useful not only for the early screening and diagnosis of IgAN but also for determining the disease severity.

## Figures and Tables

**Table 1:** Clinical characteristics of patients with IgAN and disease controls at the time of renal biopsy.

	Patients (*n*)	Age (year)	Sex	sCr (mg/dL)	eGFR (mL/min/1.73 m^2^)	UPCR (g/gCr)
Japanese						data
IgAN	338	38.1	M177/F161	0.8	83.1	0.9
DC	120	49.2	M53/F67	1.0	79.9	2.4
Korean						
IgAN	69	40.5	M28/F41	1.3	76.5	1.5
DC	39	44.4	M16/F20	1.9	69.4	4.6

IgAN, IgA nephropathy; DC, disease control; M, male; F, female; sCr, serum creatinine; eGFR, estimated glomerular filtration rate; UPCR, urinary protein-to-creatinine ratio.

**Table 2:** Clinical characteristics of disease control patients from the Japanese and Korean cohorts.

Disease Controls	Patients (*n*)	sCr (mg/dL)	eGFR (mL/min/1.73 m^2^)	UPCR (g/gCr)
Japanese cohort				
ANCA-associated glomerulonephritis	15	1.7	37.1	2.0
Lupus nephritis	26	0.7	95.7	1.7
Minimal change disease	12	0.8	87.1	5.6
Membranous nephropathy	16	0.7	90.0	3.8
Membranoproliferative glomerulonephritis	4	0.9	103.9	1.6
Non-IgA mesangial proliferative glomerulonephritis	10	0.7	101.7	0.3
Focal segmental glomerulosclerosis	5	0.9	62.3	3.2
Tubulointerstitial nephritis	10	2.0	37.2	1.7
Renal amyloidosis	6	1.0	72.1	5.1
Diabetic kidney disease	5	1.3	56.1	1.8
Nephrosclerosis	4	0.7	77.4	0.3
Thin basement membrane disease	7	0.6	119.0	0.9
Korean cohort				
ANCA-associated glomerulonephritis	12	3.9	24.9	2.3
Lupus nephritis	7	0.9	85.8	4.2
Minimal change disease	16	1.2	85.7	7.6
Thin basement membrane disease	4	0.6	108.8	0.5

sCr, serum creatinine; eGFR, estimated glomerular filtration rate; UPCR, urinary protein-to-creatinine ratio; ANCA, anti-neutrophil cytoplasmic antibody.

**Table 3:** Clinical features of patients with IgAN at the time of renal biopsy.

	Patients (*n*)	Age (year)	Sex	sCr (mg/dL)	eGFR (mL/min/1.73 m^2^)	UPCR (g/gCr)
Japanese						data
IgAN	338	38.1	M177/F161	0.8	83.1	0.9
Korean						
IgAN	69	40.5	M28/F41	1.3	76.5	1.5
Taiwanese						
IgAN	35	36.6	M21/F14	1.9	51.9	1.4

IgAN, IgA nephropathy; M, male; F, female; sCr, serum creatinine; eGFR, estimated glomerular filtration rate; UPCR, urinary protein-to-creatinine ratio.

## Data Availability

Not applicable.

## Supplementary Materials

The following supporting information can be downloaded at: https://www.mdpi.com/article/10.3390/jcm11113173/s1, Supplementary Figure S1: Levels of urinary Gd-IgA1 in Japanese, Korean and Taiwanese patients with IgAN. Levels of urinary Gd-IgA1 in Korean and Taiwanese patients with IgAN were higher than those in the Japanese patients.
