# Supplementary figures and images for: Galactose-Deficient IgA1 as a Candidate Urinary Marker of IgA Nephropathy
Source: J Clin Med. 2022 Jun 2;11(11):3173. doi: 10.3390/jcm11113173 (PMC9181435; doi:10.3390/jcm11113173)

## Slide 1
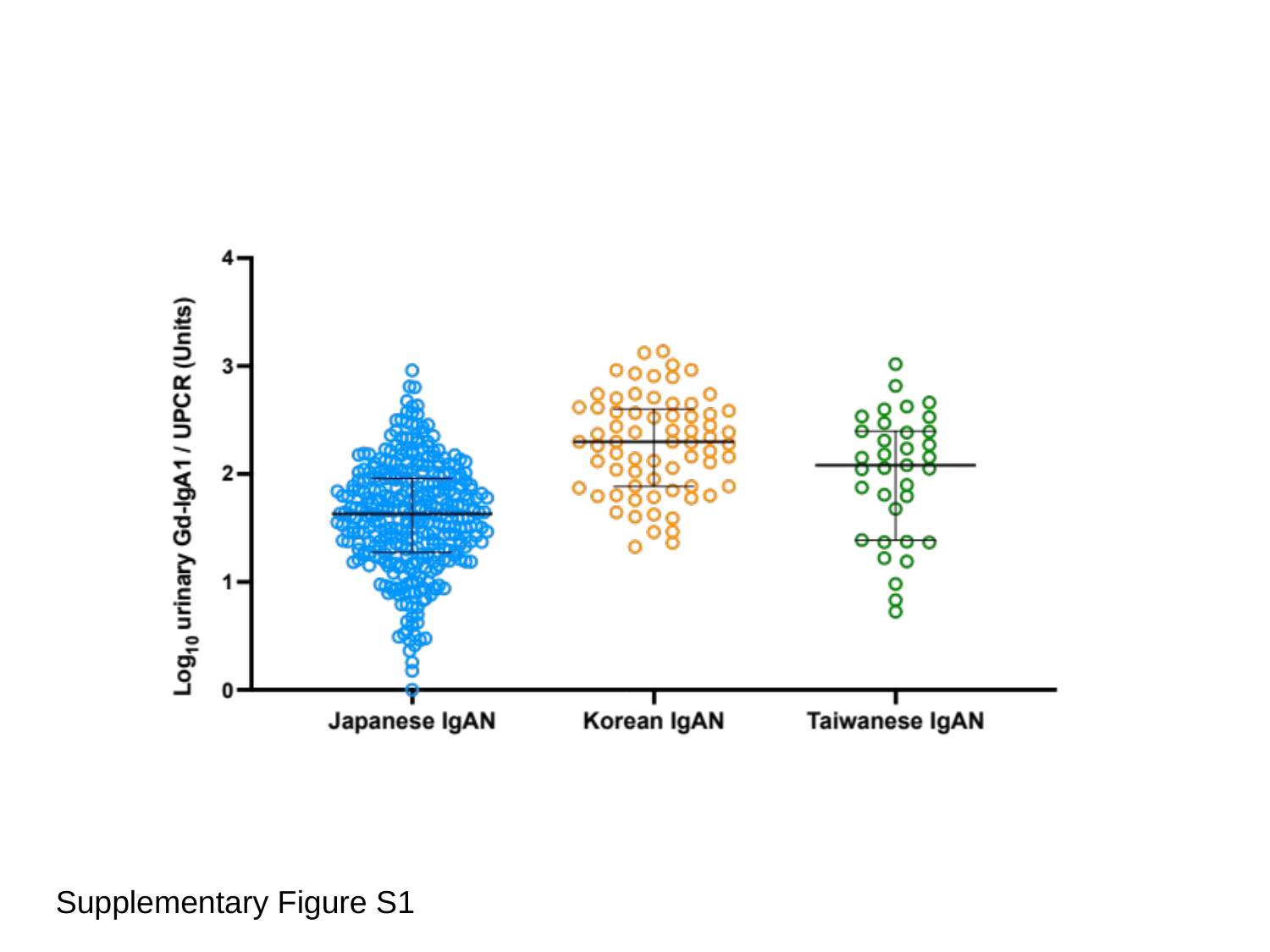

Supplementary Figure S1

Supplement: Supplementary file 1 [file jcm-11-03173-s001.zip › jcm-1684895-supplementary.pptx]
